# Green process to regenerate keratin from feathers with an aqueous deep eutectic solvent†

**DOI:** 10.1039/c9ra03305j

**Published:** 2019-06-25

**Authors:** Emmi-Maria Nuutinen, Pia Willberg-Keyriläinen, Tommi Virtanen, Alice Mija, Lauri Kuutti, Raija Lantto, Anna-Stiina Jääskeläinen

**Affiliations:** Solutions for Natural Resources and Environment, VTT, Technical Research Centre of Finland Tietotie 2 FI-02044 Espoo Finland emmi.nuutinen@vtt.fi +358406817126; Université Côte d'Azur, Université Nice-Sophia Antipolis, Institut de Chimie de Nice UMR CNRS 7272 06108 Nice Cedex 02 France

## Abstract

Poultry feathers, a source of keratin, are a significant side stream from the food industry, for which valorization is essential considering the circular economy aspects. For this, ecofriendly processes are the tools that allow the easy and feasible transformation of the feathers. Deep eutectic solvents (DESs) are generally considered as inexpensive, relatively simple, mild and environmentally friendly solvents which can dissolve proteins from protein-rich biomasses. In this work, feathers were processed with an aqueous DES to produce a uniform keratin feedstock. The proposed DES is composed of non-toxic sodium acetate and urea, with a small amount of water. After the DES treatment, water was used to dilute the DES components and regenerate the dissolved keratin. The processing conditions were optimized in terms of keratin yield and properties by varying the dissolution time from 2 h to 24 h and temperature from 80 °C to 100 °C. The yield of regenerated keratin was followed at different sodium acetate–urea molar ratios, and compared to the treatment performed with choline chloride–urea or 8 M urea as reference solvents. Sodium acetate–urea in the molar ratio of 1 : 2 at 100 °C and with 6 h dissolution time dissolved 86% of the feathers with a regenerated keratin yield of 45%. In the characterization of regenerated keratin, it was found that when the dissolution temperature was higher and the dissolution time longer, the disulfide and total sulfur content of feather keratin decreased, the range of molecular weights became wider, and some of the ordered secondary structure and crystallinity were lost.

## Abstract 2

In the present study, waste feathers were processed into uniform keratin feedstock using an aqueous, inexpensive and non-toxic deep eutectic solvent.

## Graphical abstract

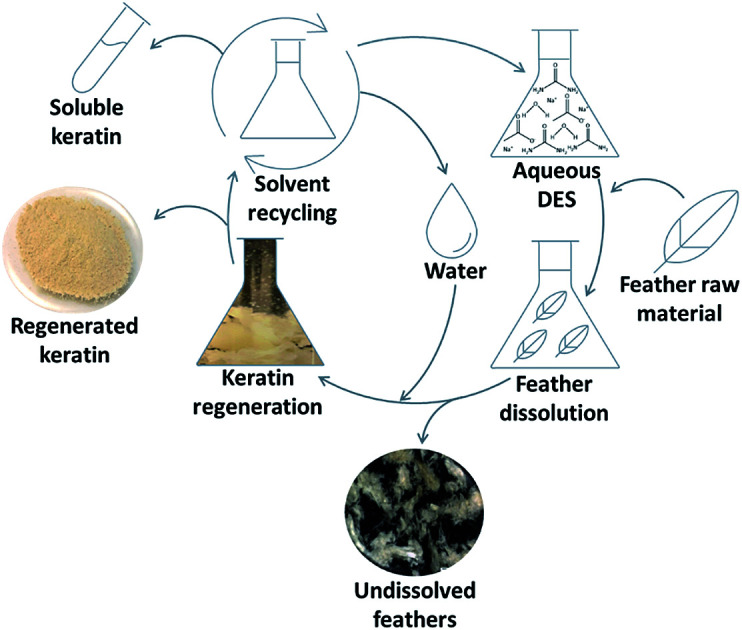

## 1. Introduction

The poultry industry generates a vast amount of waste feathers annually. In 2018, it was estimated that broiler meat production alone was going to be approximately 92 million tons,^1^ with an estimated generation of 12 million tons of feathers worldwide. These feathers are usually disposed of in landfills, incinerated or recycled as a low-value animal feed.^2^ However, feathers consist of approximately 90% of a protein, keratin. As a renewable and biodegradable biopolymer, this keratin could also be applied in a more sustainable way in value added applications. However, this kind of utilization would require conversion of the complex feather structure into a more appropriate form such as micro- and nanoparticles, fibers, films, or hydrogels.^2^ Such non-toxic, biocompatible and biodegradable keratinous bioproducts could then find commercial applications in *e.g.* the food, cosmetics, agriculture, textile, composite and medical industries.^2^

One attractive approach to obtain high quality keratin for material applications is to use specific solvents to extract the keratin from the feathers. Feather keratin is a fibrous structural protein^3^ and generally feathers are known to be insoluble in common polar and non-polar solvents due to their extensive disulfide crosslinking, high content of hydrophobic residues^4^ and tight packing of ordered secondary structures, α-helices and β-sheets, in polypeptide chains.^5^ Therefore, effective and profitable processes to extract the feather keratin are required. Usually, this extraction is done by disulfide cleavage which is achieved by reduction, oxidation, or sulfitolysis of the disulfide bonds.^6^ The chemicals which have the ability to ensure these reactions are often toxic, poorly recyclable and expensive to produce. Furthermore, the need for complex mixtures drives the need to develop more simple methods.

Ionic liquids (IL) are salts which are usually liquids at room temperature.^7^ ILs have attracted attention since they are considered as good solvents for inorganic and organic materials, they have an ability to be polar but non-coordinating solvents, they are immiscible with most organic solvents and they are non-volatile.^7^ In biomass fractionation, the dissolution with ILs is based on their capability to disrupt the hydrogen bonding within the component to be dissolved.^8,9^ ILs have also demonstrated their capacity in keratin extraction from wool^10,11^ and feathers.^12–17^

Deep eutectic solvents (DESs) are a rather new type of solvents used in biomass processing. They were first presented by Abbott *et al.*^18^ as alternative solvents for ILs. DESs are composed of solid components which together form a mixture with a melting point lower than that of its individual components.^8^ The actual DES is obtained when the components are mixed in the specific molar ratio in which the system reaches its lowest melting point.^19^ However, mixing with other molar ratios may also produce mixtures with remarkably decreased melting temperatures.^20^ The drop in the melting point is due to the hydrogen bonding between the components which decreases the lattice energy of the system.^18^ Like ILs, DESs also usually have a low vapor pressure and are non-flammable. However, unlike ILs, DESs are commonly relatively easy and inexpensive to prepare^19^ and they are usually made of non-toxic, biodegradable and biocompatible components.^20^ Probably the most cited DES, choline chloride (ChCl)–urea in the molar ratio of 1 : 2, has a melting point of 12 °C, notably lower than that of its individual components.^21^ DESs have also already demonstrated their potential in keratin extraction from wool.^11,22–24^

In this study, feather processing was investigated using a non-toxic and environmental friendly aqueous DES. The used DES was 90 wt% sodium acetate (NaOAc) – urea in the molar ratio of 1 : 2. This aqueous DES has shown great potential in protein extraction from Brewer's Spent Grain (BSG).^20^ Thermal characterization of the used DES was carried out using differential scanning calorimetry (DSC) and thermogravimetric analysis (TGA). In the feather processing, the effect of the dissolution time and temperature were studied using mass balances and different characterization methods for obtained regenerated keratin. Elemental analysis and direct colorimetric assay were used to study the disulfide, sulfhydryl and total sulfur contents, matrix-assisted laser desorption ionization time-of-flight mass spectrometry (MALDI-TOF MS) for the molecular weight range, attenuated total reflectance Fourier transform infrared spectroscopy (ATR-FTIR) and solid state nuclear magnetic resonance (NMR) for the secondary structures, X-ray powder diffraction (XRD) for the crystallinity and DSC for the thermal behavior.

## 2. Experimental

### 2.1 Materials

Sanitized chicken feathers supplied by Grupo Sada (Madrid, Spain) were ground into 2–15 mm pieces using an E-compactor (VTT, Finland) in which the feathers are pressed through a die using pan grinder rollers. Extra pure sodium acetate (NaOAc) was purchased from Honeywell (Germany), 99.0–100.6% urea from Sigma-Aldrich (Germany) and 98% choline chloride (ChCl) from Sigma-Aldrich (China).

### 2.2 Differential scanning calorimetry (DSC) and thermogravimetric analysis (TGA)

Preparation of the aqueous DES and other low melting mixtures is described in ESI.† A Mettler Toledo Differential Scanning Calorimeter (DSC820, Mettler Toledo GmbH, Switzerland) was used to perform the thermal behavior measurements. An approximately 5–15 mg sample was weighed into a pre-weighed 40 μl aluminum crucible. The pans were closed by cold pressing and holes were punctured into the lids to release any pressure arising during heating. For analysis of the melting temperatures and enthalpies of the NaOAc–urea mixtures, the samples were heated from 0 to 200 °C under N_2_ flow (80 ml min^−1^). For analysis of keratin, the samples were heated from −80 to 400 °C under N_2_ flow (80 ml min^−1^). Heating and cooling rates of 10 °C min^−1^ were used in all cases. All experiments were performed in duplicate.

Thermogravimetric analysis (TGA) of the 1 : 2 NaOAc–urea mixtures were carried out using a Netzsch device (STA449F1, Netzsch Gerätebau GmbH, Germany) under N_2_ flow (40 ml min^−1^). The analyzing temperature range was from 40 to 1000 °C with a heating rate of 5 °C min^−1^.

### 2.3 Elemental analysis

Elemental analysis (C, H, N and S) was carried out using a FLASH 2000 Organic Elemental Analyzer (Thermo Scientific, Germany). Before the analysis, samples were dried overnight in an oven at 105 °C in order to remove excess moisture. The average values of two duplicate samples were measured and reported.

### 2.4 Protein content

Total protein content can be approximated by multiplying the nitrogen content by a factor corresponding to the average nitrogen content of proteins. The nitrogen content of the native feathers was determined by elemental analysis and the protein content was then determined using 6.66 as a conversion factor.^25^ The soluble protein content was determined with a two-step method. First, diluted aqueous samples were precipitated using the commercial Compat-Able™ Protein Assay Preparation Reagent Set (Thermo Scientific, USA).^26^ Then the protein content was measured using the commercial BCA protein assay kit (Thermo Scientific, USA).^27^

### 2.5 Sulfur compounds

The direct colorimetric solid phase assay for free sulfhydryl groups and disulfide bonds described by Chan and Wasserman^28^ was used to determine the sulfhydryl (SH) and disulfide (S–S) contents in the native feathers and in the regenerated keratin samples. Water was used to dilute samples when needed. 2-Nitro-5-thiosulfobenzoate (NTSB^2−^) needed in the assay was synthesized according to Thannhauser *et al.*^29^ The concentrations were calculated using 13 600 M^−1^ cm^−1^ as an extinction coefficient and total sulfhydryl content was assumed to be given by the sum of [SH] + 2[S–S]. Between 2–4 replicates were measured and the average value of each sample was reported.

### 2.6 Matrix-assisted laser desorption ionization time-of-flight mass spectrometry (MALDI-TOF MS)

Matrix-assisted laser desorption ionization time-of-flight mass spectrometry (MALDI-TOF MS) was applied to determine the ranges of molecular weights of the keratin samples. Before the measurement, keratin samples were dissolved in a mixture of 1.5% dithiothreitol (DTT), 0.5 M tris hydrochloride (HCl), 10% glycerol and 2% sodium dodecyl sulfate (SDS). Sinapinic acid was selected as the matrix and dissolved to saturation in a mixture of 0.1–0.3% trifluoroacetic acid (TFA) and 50% acetonitrile. One microliter of the matrix mixture and sample were placed on the target plate and dried under air. The analysis was conducted using a Bruker mass spectrometer Autoflex II Maldi-TOF LRF50-CID (Bruker Daltonik GmbH, Germany).

### 2.7 Fourier transform infrared spectroscopy (FTIR)

A Fourier transform infrared spectroscopy (FTIR) spectrometer equipped with an attenuated total reflectance (ATR) diamond crystal (Nicolet iS50, Thermo Scientific, USA) was used for the structural studies. All spectra were collected using 32 scans in a spectral range of 4000–400 cm^−1^ and with a spectral resolution of 4 cm^−1^. Several spectra were collected from different locations of each sample, and the average of these spectra was used in the analysis. The spectra were processed with OriginPro 2017 software. Deconvolution of the bands was carried out using OriginPro 2017 software (Multiple Peak Fit) and Gaussian fit following the studies of Tsuboi *et al.*^30^ and Rintoul *et al.*,^31^ and the bands for each secondary structure were assigned according to the studies of Barth^32^ and Miyazawa & Blout.^33^ Deconvolutions were carried out for three or four spectra of each sample, and the average and standard deviation were reported.

### 2.8 Nuclear magnetic resonance spectroscopy (NMR)

The ^13^C cross polarization (CP) magic angle spinning (MAS) Nuclear magnetic resonance spectroscopy (NMR) measurements were performed using an Agilent DD2 600 spectrometer (Agilent Technologies, USA), with magnetic flux density of 14.1 T and equipped with a 3.2 mm T3 MAS NMR probe operating in double resonance mode. The MAS rate in experiments was 10 kHz, the number of scans was 10 000 with a 6.0 s delay between successive scans, and CP contact time was 1.3 ms. The spectra were processed using TopSpin 3.5 and OriginPro 2017 software. Deconvolution of the peak was carried out using OriginPro 2017 software (Multiple Peak Fit) and Gaussian functions. Deconvolution was performed based on the studies of Duer *et al.*^34^ and Idris *et al.*^12^

### 2.9 X-ray powder diffraction (XRD)

X-ray powder diffraction (XRD) was used to determine the semi-crystallinity of feather keratin samples. A Philips X'pert Pro diffractometer (PW 1130/00, Philips, The Netherlands) was used with Ni-filtered Cu Kα radiation (*λ* = 1.541 Å). The data were obtained with geometry in which the sample was kept in place and the tube and counter were in motion. The X-ray beam was generated at 45 kV and 30 mA. Diffraction intensities were recorded with 2*θ* ranging from 4° to 60° at a step size of 0.079° and a scan step time of 390 s. The data was processed using X'Pert and OriginPro 2017 software. Deconvolution of the peaks was carried out using OriginPro 2017 software (Multiple Peak Fit) and Gaussian functions. Deconvolution was performed based on the study of Cao & Billows.^35^

### 2.10 Dissolution and regeneration of feather keratin

Regenerated keratin samples were obtained by dissolving the feathers in the aqueous DES after which the dissolved keratin was regenerated in water. 3 g of ground feathers were added in 147 g of preheated (80–100 °C), clear, freshly prepared solvent. The used solvents were 90 wt% NaOAc–urea in the molar ratio of 1 : 2 and 1 : 3. 90 wt% ChCl–urea in the molar ratio of 1 : 2 and 8 M urea were used as reference solvents for NaOAc–urea. The solution was mixed from 2 h to 24 h. After the desired dissolution time, the solution was filtered through a metallic wire mesh (90 μm) in a heated (105 °C) and pressurized (3–5 bar) filtration unit. The solid residue was washed with Milli-Q water and freeze-dried. The filtrate was added to 350 ml of Milli-Q water to regenerate the dissolved feather keratin and dissolve the DES components. Regenerated keratin was then filtered in a Büchner funnel (60 μm), washed with Milli-Q water and freeze-dried.

## 3. Results and discussion

### 3.1 Preparation and characterization of the aqueous DES and low melting mixtures

In this study, urea was coupled with NaOAc to form a mixture which had a lower melting point than urea and NaOAc alone. The DSC results (Table S1 and Fig. S1†) indicate that the lowest melting temperature (85 °C) was achieved for the mixture of NaOAc and urea in the molar ratio of 1 : 2. Based on this result, it was decided to use this ratio as DES in this study. In earlier studies, NaOAc trihydrate–urea mixtures are reported to reach their eutectic point (30 °C and 33 °C) in the molar ratio of 2 : 3.^36,37^ However, in these studies, it should be noted that the portion of water changes along with the portion of NaOAc trihydrate, and in DES systems, water decreases the melting point. This is due to the capability of water to decrease the lattice energy of the mixture through new hydrogen bonds.^19^ To speed up the melting and decrease the viscosity of the solution without completely destroying the hydrogen bonding between the DES components, 10 wt% of water was added to the 1 : 2 NaOAc–urea mixture. In this way the melting temperature decreased from 85 °C to 38 °C (Table S1†). TGA was carried out to identify the suitable processing temperature of the chosen mixture. Because the loss of water can be controlled with the cooling system in the process, it is suggested that the processing temperature of this aqueous DES should be between 38 °C and 105 °C in order to avoid the decomposition of DES components (Fig. S2†).

### 3.2 Dissolution and regeneration of feather keratin

**Fig. 1:**
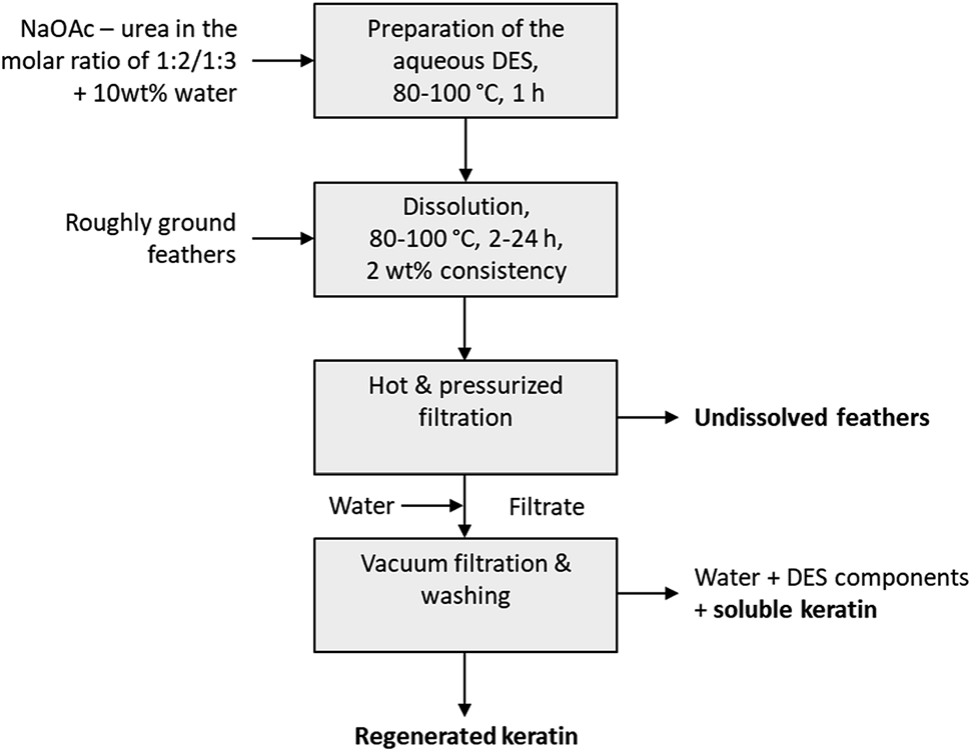
Operational procedure for feather treatment with an aqueous DES.

When the feathers were processed with the aqueous DES, three different fractions were obtained: undissolved feathers, regenerated keratin, and soluble keratin (Fig. 1). After the separation of the undissolved feathers with heated and pressurized filtration system (90 μm), the dissolved keratin regenerated into water. Moreover, not all of the dissolved keratin precipitated in the regeneration step, but rather dissolved in water together with NaOAc and urea. This fraction is called soluble keratin. The success of the processing was studied using the mass balances. The gravimetric yields of undissolved feathers and regenerated keratin were obtained by weighing the freeze-dried samples. The soluble keratin content was determined with a BCA protein assay kit. In this study, an unidentified part is also reported. This fraction corresponds to the part of the applied feathers (3 g) which was not captured when mass balances were calculated.

**Fig. 2:**
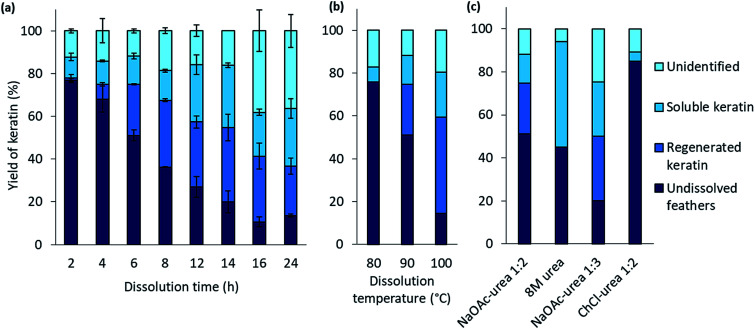
The yields of feather keratin in each fraction using 90 wt% 1 : 2 NaOAc–urea (a) at 90 °C for 2–24 h, (b) at 80–100 °C for 6 h, and (c) using 8 M urea, 90 wt% 1 : 3 NaOAc–urea and 90 wt% 1 : 2 ChCl–urea at 90 °C for 6.

The effect of the dissolution time in the feather processing was investigated using a dissolution temperature of 90 °C and varying the time from 2 h to 24 h. The time series at 90 °C was performed in duplicate to illustrate the standard error. The results (Fig. 2a) show that when the dissolution time increased from 2 h to 14 h, the yield of regenerated keratin increased from 1% to 35% and that of soluble keratin from 10% to 21%, while the undissolved feather fraction decreased from 77% to 20%. When the dissolution time was further increased to 16 h and 24 h, the yield of undissolved feather fraction continued to decrease down to 11%, and regenerated and soluble keratin also started to decrease. On the other hand, the unidentified fraction increased to 38%. Based on these results, it appears that in feather processing using the aqueous DES, the dissolution of feathers as well as the generation of regenerated and soluble keratin occurred concurrently. However, when a certain level of regenerated keratin was reached, the growth of soluble and unidentified fractions appeared to be the main phenomenon. This could indicate the cleavage of protein chains into low molecular weight components such as polypeptides or amino acids.

The effect of the dissolution temperature was investigated using 6 h dissolution time and varying the dissolution temperature from 80 °C to 100 °C. Fig. 2b represents the results. It can be detected that when the temperature was increased from 80 °C to 100 °C, the yield of regenerated keratin increased from 0% to 45% and that of soluble keratin from 7% to 21% whereas the fraction of undissolved feathers decreased from 83% to 14%. It is clear that temperature has an important effect in the dissolution. It is suggested that at higher temperature the dissolution is more effective due to lower viscosity of the solvent and higher mobility of the solvent components compared to the lower temperatures.^38^ When the viscosity of the system decreases, the mass transfer and diffusion of DES components into the structures are enhanced.^23,39^ The keratin yields obtained at 100 °C and with 6 h dissolution time are comparable to some other feather processing methods. In this case, 86% of feathers were dissolved in the aqueous DES. In previous studies, Sharma *et al.*^40^ and Yin *et al.*^41^ have obtained keratin yields of 80% and 90% for acid precipitated keratin, respectively. Sharma *et al.*^40^ dissolved feathers in 0.5 M sodium sulfide while in the study of Yin *et al.*^41^ feathers were processed with Shindai method in which feathers are treated with ethanol pretreatment, hydrochloric acid pretreatment and 2-mercaptoethanol.

**Fig. 3:**
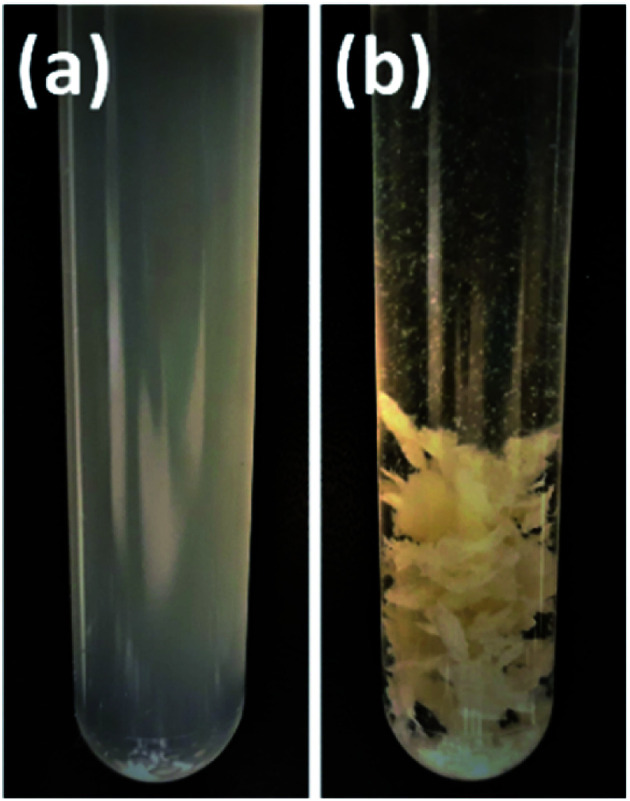
Samples after processing with (a) 8 M urea and (b) aqueous DES (1 : 2 NaOAc–urea) when water was added to the solution to regenerate the keratin.

In protein denaturation, the role of urea as a denaturant is well established. It has also been used in feather keratin extraction with other chemicals such as reducing agents.^4,42–44^ It is known that urea has an ability to unfold proteins.^45^ As a reference to the aqueous DES, the effect of 8 M urea was studied to dissolve feathers at 90° for 6 h. As expected, the results (Fig. 2c) show that 8 M urea was also able to dissolve 55% of the feathers. However, with urea, none of the desired fraction, regenerated keratin, was observed. The difference is visualized in Fig. 3. With 8 M urea, keratin did not start to precipitate before the pH of the solution was adjusted to 4.6. This kind of additional step could further complicate the recycling of the solvent. It is clear that the behavior of urea in DES differs from its behavior in aqueous solutions due to the strong interactions between the DES components. The effect of urea in the NaOAc–urea mixture was also studied by increasing to portion of urea in a way that the molar ratio of the aqueous NaOAc–urea mixture was 1 : 3. It was found that this molar ratio was able to dissolve 80% of the feathers with the formation of 30% of regenerated keratin at 90 °C for 6 h which was more than with the molar ratio of 1 : 2 or 8 M urea (Fig. 2c). Thus, the yields of different fractions can be tailored not only by tuning the dissolution conditions but also the molar ratio of NaOAc–urea mixture.

The other main component in the aqueous DES is composed of sodium cation and acetate anion (NaOAc). It is suggested that in some DES systems, salts may act as stabilizing agents for proteins as they increase the ionic strength of the environment.^46^ It has also been suggested that the protein can remain stable in some DES systems because the hydrogen bonding between urea and anion limits the penetration of urea molecules deep into the protein core, and DES components preferably interact with the surface residues of protein.^47^

When 90 wt% DES consisting of ChCl and urea in the molar ration of 1 : 2 was tested for the feather dissolution at 90 °C for 6 h, no regenerated keratin was formed and the undissolved fraction was 87% (Fig. 2c). In addition to urea, the role of anion is suggested to be important in the dissolution processes with DESs.^22^ Moore *et al.*^22^ suggested that wool dissolution in ChCl–urea is due to the capability of highly polar anion and urea to disturb the hydrogen bonding, the disulfide and electrostatic bonds present in the wool. It has been suggested that because the acetate anion has higher basicity than chloride, it could more effectively disturb the hydrogen bonding within the material.^48^ The different cation may also partly explain the difference between the aqueous DESs. However, the effect of cations is suggested to be less important than that of anions.^22^

A small addition of water is also proposed to have a positive effect on the dissolution with NaOAc–urea DES, as it decreases the viscosity of the mixture, thus facilitating the mass transfer and in this way the dissolution of the system.^20^ However, it should be taken into account that too much water can be harmful because it starts to compete with the hydrogen bonding which is responsible for the dissolution.^9^

### 3.3 Characterization of regenerated keratin

#### 3.3.1 Sulfur compounds

**Fig. 4:**
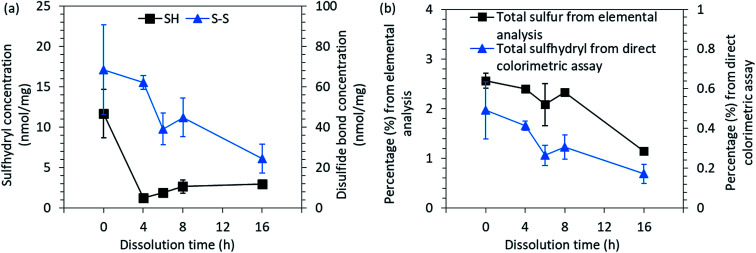
(a) The concentration of free sulfhydryl groups (SH) and disulfide bonds (S–S) and (b) the total sulfur and sulfhydryl contents present in the native feathers and in regenerated keratin.

Feather keratin possesses disulfide bonds that restrict its dissolution. In feather keratin, sulfur is located in disulfide bonds (S–S), in sulfhydryl groups of cysteine or in methionine.^4^ Because the content of methionine is considered to be low,^4^ the sulfur is almost completely located in disulfide bonds and sulfhydryl groups. In order to understand the dissolution with aqueous DES, the disulfide, free sulfhydryl and total sulfur contents were studied with the direct colorimetric assay and elemental analysis. The results (Fig. 4) indicate that the DES treatment decreased the concentration of disulfide bonds from 68.5 nmol mg^−1^ to 24.5 nmol mg^−1^ when the dissolution time was increased from 0 h to 16 h. A similar trend has been observed in other studies in which feathers were treated by steam explosion.^49,50^

After the cleavage of one disulfide bond, two cysteine residues are formed. Between the regenerated keratin samples, an increasing trend of sulfhydryl groups could be observed towards the longer dissolution time as the concentration increased from 1.3 nmol mg^−1^ to 3.0 nmol mg^−1^ between the 2 h and 16 h dissolution times (Fig. 4a). However, the free sulfhydryl group contents in regenerated keratin samples were lower than in the native feathers (11.7 nmol mg^−1^). The decrease in the sulfhydryl content indicates the destruction of cysteine residues and the further reactions of the free sulfhydryl groups into sulfur-containing volatiles during the processing.^49^ The loss of sulfur can also be seen in Fig. 4b, in which total sulfur and sulfhydryl contents measured by elemental analysis and colorimetric assay are presented. Both analyses show a similar decreasing trend in sulfur content. However, a difference can be seen in the percentages. The difference between the methods can probably be explained by the accessibility to the structure. In elemental analysis, the structure of the sample was destroyed and all the sulfur could be detected, whereas in the colorimetric assay, the color reagents may not have been able to react with all the sulfhydryl groups because not all the keratin was dissolved in the reaction buffer. However, although the colorimetric assay may not give the absolute values, a common trend could be discerned.

#### 3.3.2 Molecular weight

**Fig. 5:**
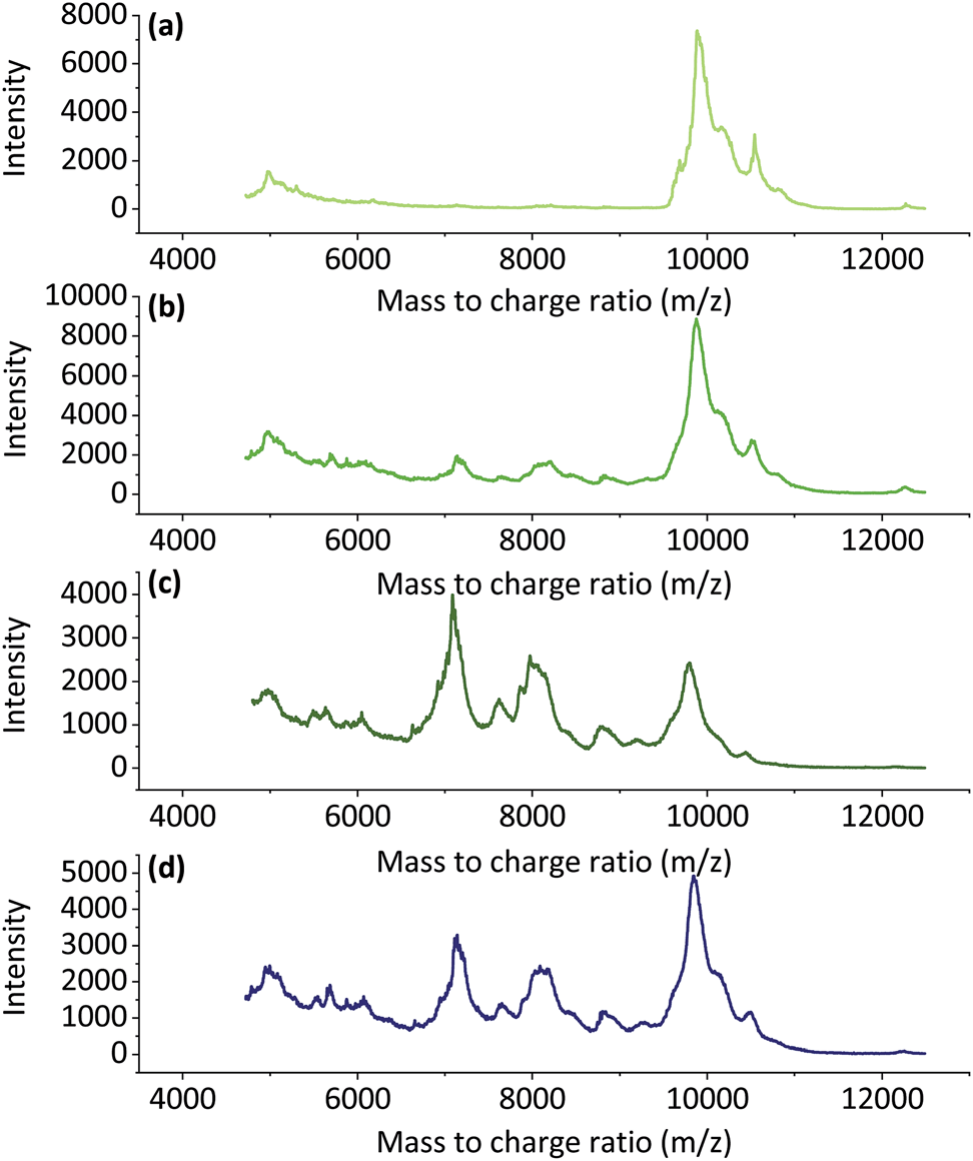
The MALDI-TOF spectra for regenerated keratin at 90 °C for (a) 4 h, (b) 8 h, (c) 16 h and (d) at 100 °C for 6 h.

MALDI-TOF MS was used to study changes in the molecular weights of the regenerated keratin samples. In the literature, it is proposed that the molecular weights of feather keratin is around 10 kDa.^51^ The native feathers could not be dissolved completely and therefore no reliable MALDI-TOF spectra could be measured. Fig. 5, which represents the data obtained from MALDI-TOF MS, shows that with longer dissolution time (Fig. 5a–c) and with higher dissolution temperatures (Fig. 5d), the range of molecular weights became wider. After 4 h dissolution time, a wide peak can be observed around 10 000 *m*/*z* and a smaller wide peak at approximately 5000 *m*/*z*. When the dissolution time and temperature increased, wide peaks started to appear at approximately 7000 *m*/*z* and 8000 *m*/*z*, and peak at 10 000 *m*/*z* started to decrease. This could indicate degradation of the keratin chains. This supports the conclusion that the increase in the soluble keratin yield and the loss in total yield were due to degradation of the polypeptide backbones.

#### 3.3.3 Secondary structures

Feather keratin presents two different ordered conformations in its secondary structure. The polypeptide chain can either curl into α-helices or bond into plated sheets, β-sheets. In addition to these ordered structures, feather keratin includes disordered structures, random coil, and chain reversal regions, β-turns, between the β-sheets.^52^

**Fig. 6:**
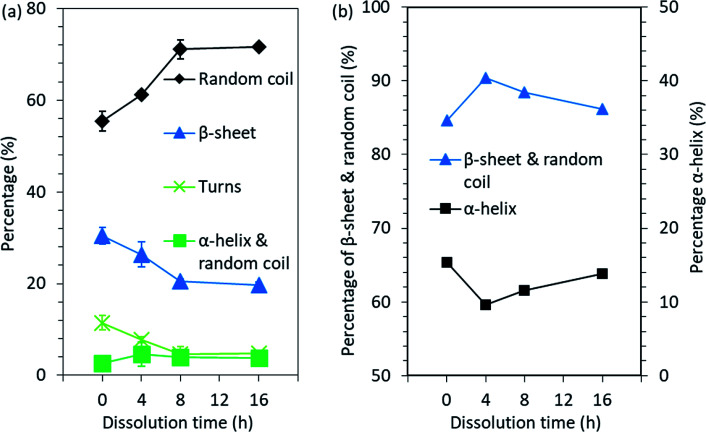
Changes in the secondary structure of feather keratin after the processing obtained from (a) the FTIR spectra band fitting and (b) the NMR spectra peak fitting.

ATR-FTIR was used to study the chemical structure and more specifically the secondary structures of the feather keratin. Although MALDI-TOF MS indicated that the keratin molecules started to degrade during the DES processing, the FTIR spectra (Fig. S3†) suggest that most of the polypeptide backbone in the regenerated keratin was retained. This can be observed from the shape of the spectra and especially from the amide I and II bands. The amide I band at 1600–1700 cm^−1^ is mainly assigned to the C=O stretching vibration, while the amide II band at 1480–1570 cm^−1^ originates from out-of-phase combination of NH bending and CH stretching vibration.^32^ The amide I–II vibrations are known to be less affected by the nature of the side chains but rather by the conformation of the polypeptide backbone.^32^ Therefore, these bands could also be used to study the secondary structures of proteins. In order to investigate the secondary structures more closely, the overlapping bands present in the amide I–II bands were determined by the deconvolution of these bands in their individual components (Fig. S4†). The results obtained from band fitting are summarized in Fig. 6a. The data obtained from the deconvolution shows that when the dissolution time was increased from 0 h to 16 h, the disordered structure, random coil, increased from 55.4% to 71.6% and the portion of α-helix & random coil increased from 2.6% to 3.9%, while the percentage of β-sheet and turns decreased from 30.5% and 11.5% to 19.7% and 4.8%, respectively. The data and results obtained from the deconvolution should be treated with caution, as the parameter choice is rather subjective. However, although the values obtained should not be interpreted as absolute, a common trend can be concluded.

In addition to the ATR-FTIR, NMR was also used to investigate the molecular conformations and dynamics in the feather keratin. The spectra obtained (Fig. S5†) are characteristics for the feather keratin,^12^ and are similar to each other with slight differences in the intensities, especially in the alkyl and α-carbon region (10–70 ppm). This indicates that no major changes in the protein backbone, but rather some changes in the conformation and side chains, occurred during the processing.^34^ The peak at 176 ppm is assigned to carbonyl carbons, and these carbons give slightly different shifts depending on whether they are located in α-helix or β-sheet.^34^ Usually, α-helix gives a shift at higher ppm than β-sheet.^34^ To detect the overlapping peaks and different secondary structures in the peaks in the 176 ppm region, deconvolution was carried out (Fig. S6†). The results are summarized in Fig. 6b. When the native feathers are compared to the regenerated keratin sample which was treated at 90 °C for 4 h, the percentage of β-sheet and random coil increased from 84.6% to 90.4%, while α-helix decreased from 15.4% to 9.6%. With longer dissolution time, the portion of β-sheet and random coil started to decrease and α-helix to increase, and after 16 h the percentage of β-sheet and random coil was 86.2% and that of α-helix was 13.8%. The discussion of the comparison between the results obtained from NMR and ATR-FTIR is difficult as NMR gives the percentage of β-sheet and random coil together and α-helix separately while in ATR-FTIR the percentages of β-sheet and random coil are given separately and α-helix and random coil together. However, based on both methods, it seems that some of the ordered secondary structure was lost during the processing.

The changes of secondary structures was also noticed in DSC studies (Fig. S7†). In the native feathers the endothermic peak at 233 °C indicates the melting of α-helices.^53,54^ For regenerated keratin samples this peak appeared already at 223 °C and is broadened compared to the native feathers. The shift towards lower temperatures and the change in the shape may indicate a greater portion of disordered structure^12^ or higher moisture content^53^ in the regenerated keratin compared to the native feathers.

#### 3.3.4 Crystallinity

**Fig. 7:**
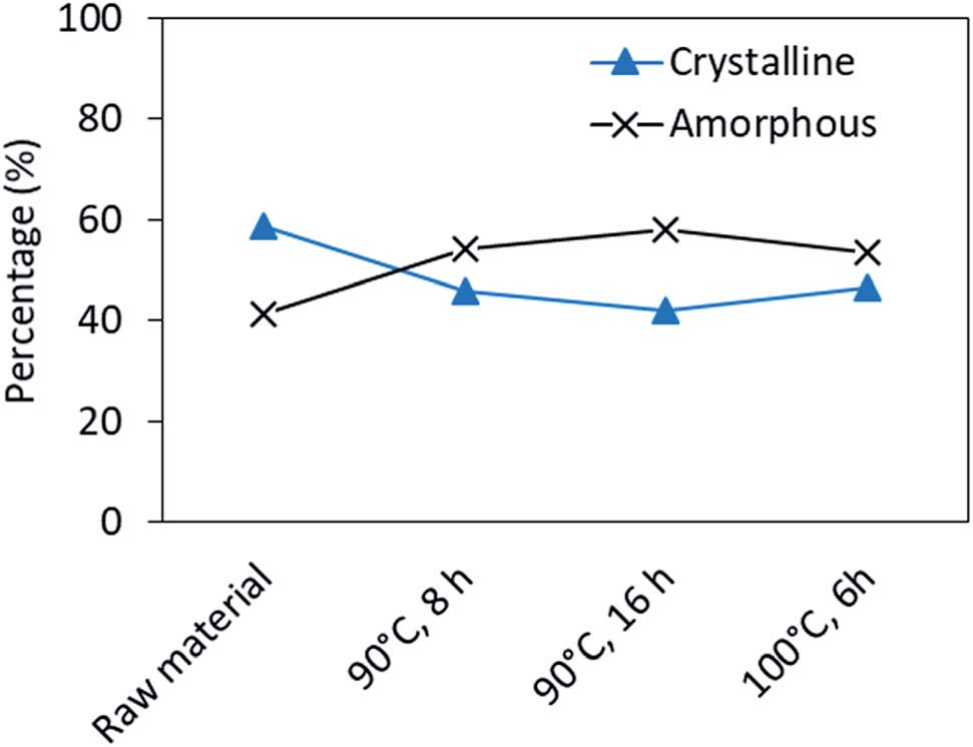
Changes in the crystallinity of regenerated keratin after processing, obtained from the deconvolution of XRD patterns.

As well as the disulfide bonding, the crystallinity is also believed to be an important factor in keratin's strength and stiffness.^50^ XRD was used to study and compare the crystallinity of the native feathers and regenerated keratin samples. Fig. S8† represents the XRD patterns obtained. The patterns clearly indicate that all the samples were semi-crystalline keratin, with most notable peaks at approximately 9° and 20°. In the native feathers, the peak at 9° is broader than in regenerated keratin samples and starts already at approximately 4°. Furthermore, a small and broad peak at 40° can be observed. On the other hand, in the processed keratin samples, new small sharp peaks can be observed. Different spacing as well as peak positions in XRD patterns indicate different structures and arrangements of crystals.^55^ In regenerated keratin samples, these new peaks most probably indicate new crystalline structures which appeared during regeneration. The most significant peaks at 9° and 20° correspond to the crystalline spacing of 9.8 Å and 4.4 Å, respectively.^55^ In all samples, the peaks at 9° have higher intensity than those at 20°, and this difference is greatest in the native feathers. The diffraction spot at 5.1 Å (2*θ* = 17.8°) is associated with α-helix, the spot at 4.65 Å (2*θ* = 19°) with β-sheet, and the spot at 9.8 Å (2*θ* = 9°) with both α-helix and β-sheet.^35,56^ Due to the tight overlapping of α-helix and β-sheet, their unambiguous assignment to individual components is not possible. However, deconvolution of crystalline and amorphous intensity profiles could be carried out in order to determine the portion of crystallinity in the samples (Fig. S9†).^35^ The results (Fig. 7) indicate that the degree of crystallinity decreased from 58.5% to 42.0% after 16 h of DES processing. The loss of the crystalline and ordered secondary structures is most probably related to the cleavage of disulfide bonds and polypeptide chains as well as to the change of interactions like hydrogen bonding present in feather keratin.

## 4. Conclusions

In the present study, the processing of waste feathers using an aqueous, inexpensive and food-grade DES was successfully demonstrated. It is suggested that the processing of feathers with aqueous DES is a complex system in which urea, sodium acetate as well as water take part. Regenerated keratin, which could be further applied in biomaterials, was obtained by precipitating the dissolved feather in water. In the characterization process of the regenerated keratin, it was observed that no major chemical changes occurred in the polypeptide backbone, and the part which precipitated and regenerated into water had the ability to either retain or rearrange part of its ordered and crystalline structure. The dissolution of keratin and the structural differences of regenerated keratin are proposed to be due to the ability of the aqueous DES to disturb the interactions within the feather keratin, cleave the disulfide bonds and partly break down the polypeptide backbone of keratin. However, although some structural differences took place after the aqueous DES processing, this method is proposed to represent a rather gentle and, with further optimization, potential processing method in which the properties as well as the yields of different fractions can be tailored by tuning the dissolution conditions and molar ratio of DES.

## Conflicts of interest

There are no conflicts to declare.

## Supplementary Material

RA-009-C9RA03305J-s001
