# Supplementary material for: Green process to regenerate keratin from feathers with an aqueous deep eutectic solvent
Source: RSC Adv. 2019 Jun 25;9(34):19720–8. doi: 10.1039/c9ra03305j (PMC9065387; doi:10.1039/c9ra03305j)
Supplement: RA-009-C9RA03305J-s001 [file RA-009-C9RA03305J-s001.pdf]

Electronic Supplementary Material (ESI) for RSC Advances.
This journal is © The Royal Society of Chemistry 2019

Electronic supplementary information (ESI)

Green process to regenerate keratin from feathers with an aqueous deep eutectic solvent

Emmi-Maria Nuutinen†, Pia Willberg-Keyriläinen†, Tommi Virtanen†, Alice Mija‡, Lauri Kuutti†, Raija Lantto†, Anna-Stiina Jääskeläinen†*

† Solutions for Natural Resources and Environment, VTT, Technical Research Centre of Finland, Tietotie 2, FI-02044, Espoo, Finland

‡ Université Côte d'Azur, Université Nice-Sophia Antipolis, Institut de Chimie de Nice, UMR CNRS 7272, 06108 Nice Cedex 02, France

* Corresponding author: E-mail: emmi.nuutinen@vtt.fi. Telephone: +358406817126.

Preparation of the aqueous DES and low melting mixtures

The aqueous DES and other low melting mixtures were prepared from extra pure sodium acetate (NaOAc) (Honeywell, Germany) and urea (99.0-100.6 %, Sigma-Aldrich, Germany) by mixing the solid components in the specific molar ratios with or without water (10 % by weight) in cap sealed glass vials at ~ 150 °C under continuous stirring (100 rpm) until a clear solution was obtained. Samples were allowed to cool down and recrystallize before analysis. Differential scanning calorimetry (DSC) was used to determine the melting temperatures and enthalpies of these mixtures with different NaOAc - urea molar ratios (1:0, 5:1, 2:1, 1:2, 1:3, 1:5, and 0:1).

Dissolution and regeneration of feather keratin

Table S1 Melting points and enthalpies of NaOAc - urea mixtures.

Molar ratio of NaOAc - urea	Water content	Melting temperature (°C)*	Melting enthaply (J/g)*
1:0	No added water	328 ± 0.23	220 ± 1.94
5:1	No added water	58 ± 6.24**	11 ± 3.48**
2:1	No added water	86 ± 1.40**	33 ± 0.21**
1:2	No added water	85 ± 0.74	70 ± 4.34
1:3	No added water	101 ± 0.76	130 ± 0.43
1:5	No added water	103 ± 0.01	168 ± 7.24
0:1	No added water	134 ± 0.01	244 ± 1.16
1:2	With 10 wt% water	38 ± 0.78	33 ± 24.13

* 1st heating cycle ** Did not melt completely

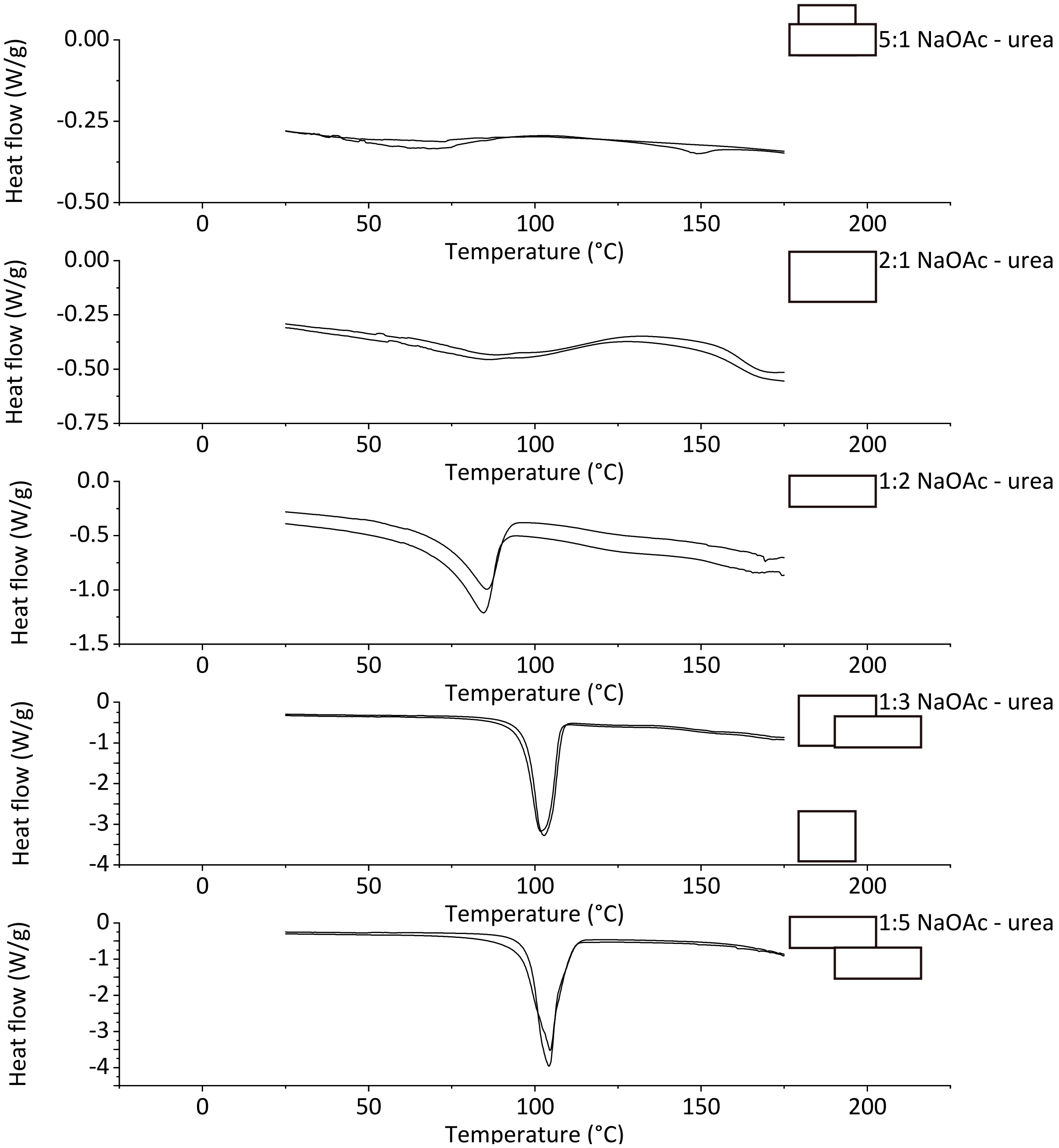

Fig. S1 DSC traces for NaOAc - urea mixtures.

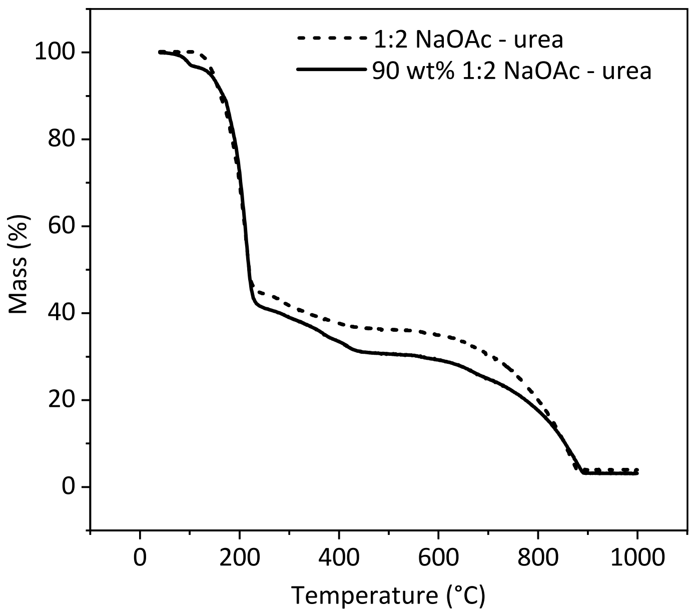

Fig. S2 TGA thermogram under N_2 at 5 °C/min for dry and 90 wt% 1:2 NaOAc - urea.

Characterization of regenerated keratin

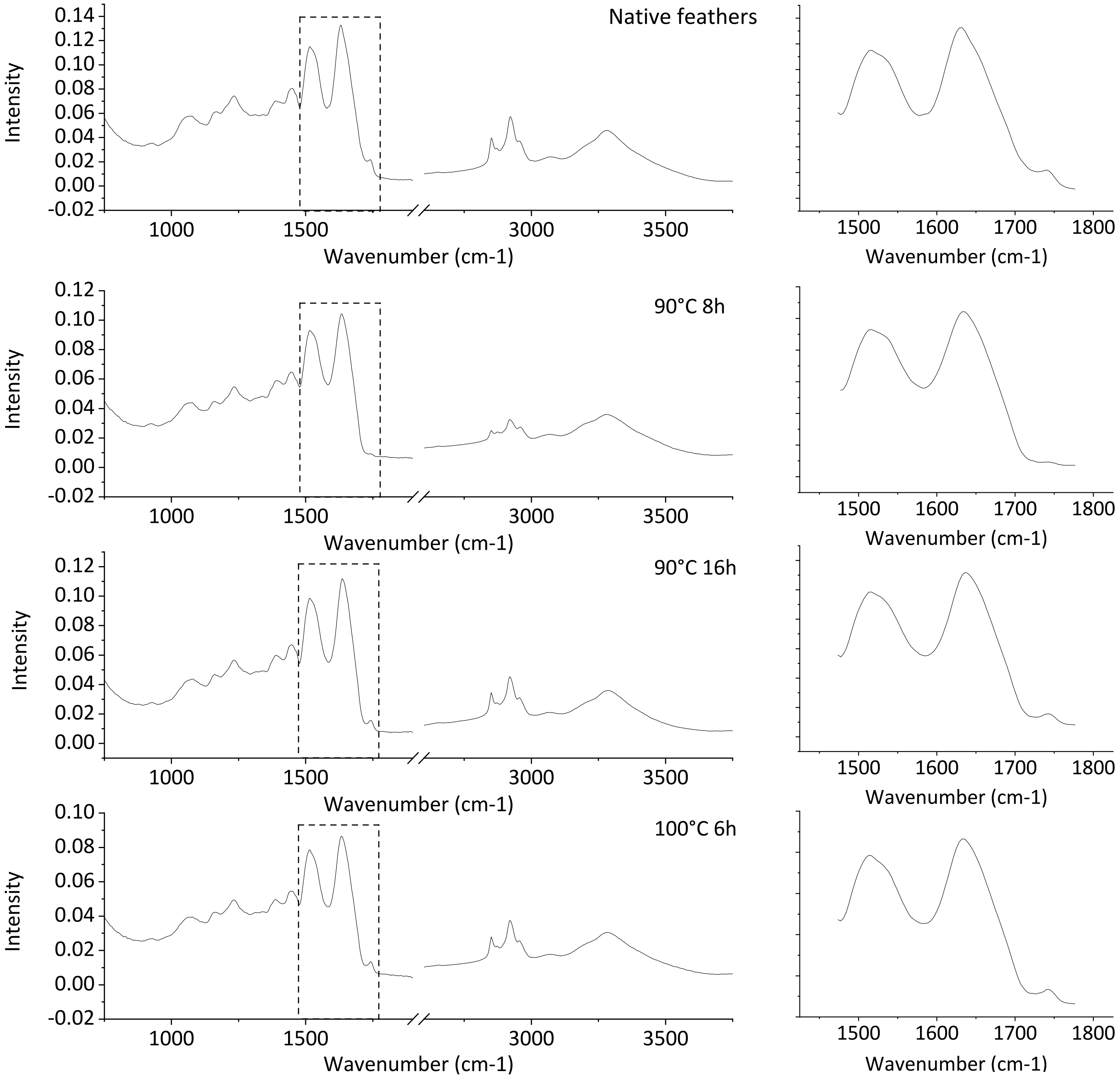

Fig. S3 ATR-FTIR spectra of the raw material and regenerated keratin samples.

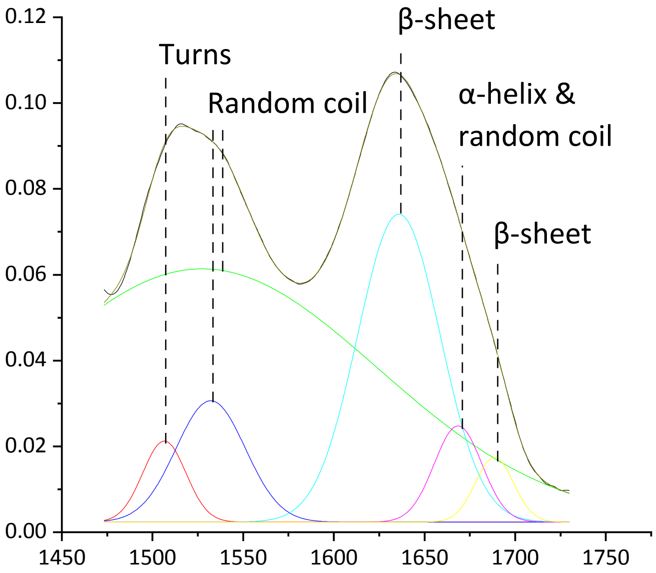

Fig. S4 Band fitting in ATR-FTIR spectrum.

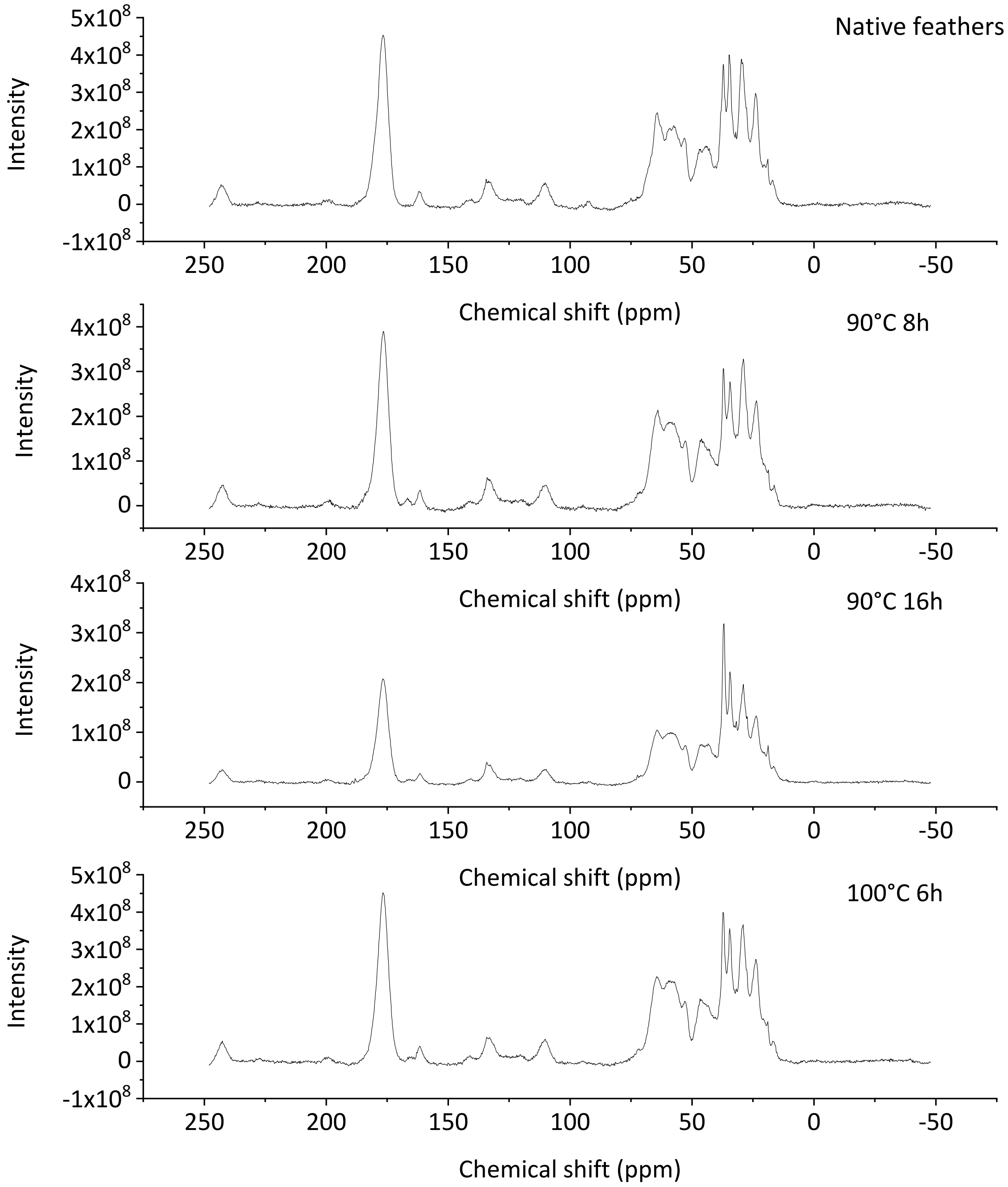

Fig. S5 NMR spectra of the raw material and regenerated keratin samples.

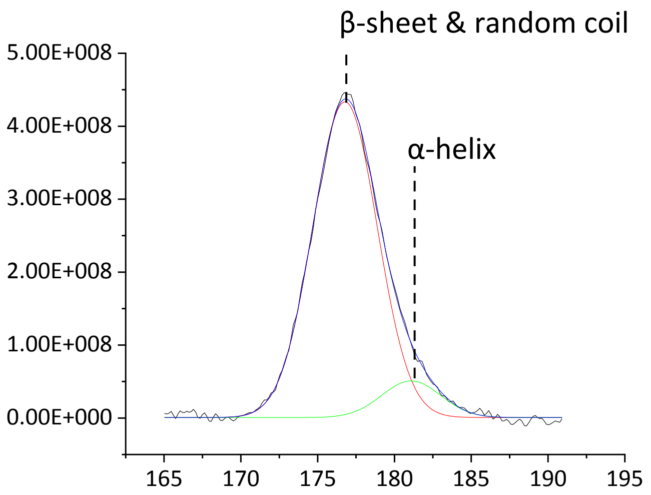

Fig. S6 Peak fitting in NMR spectrum.

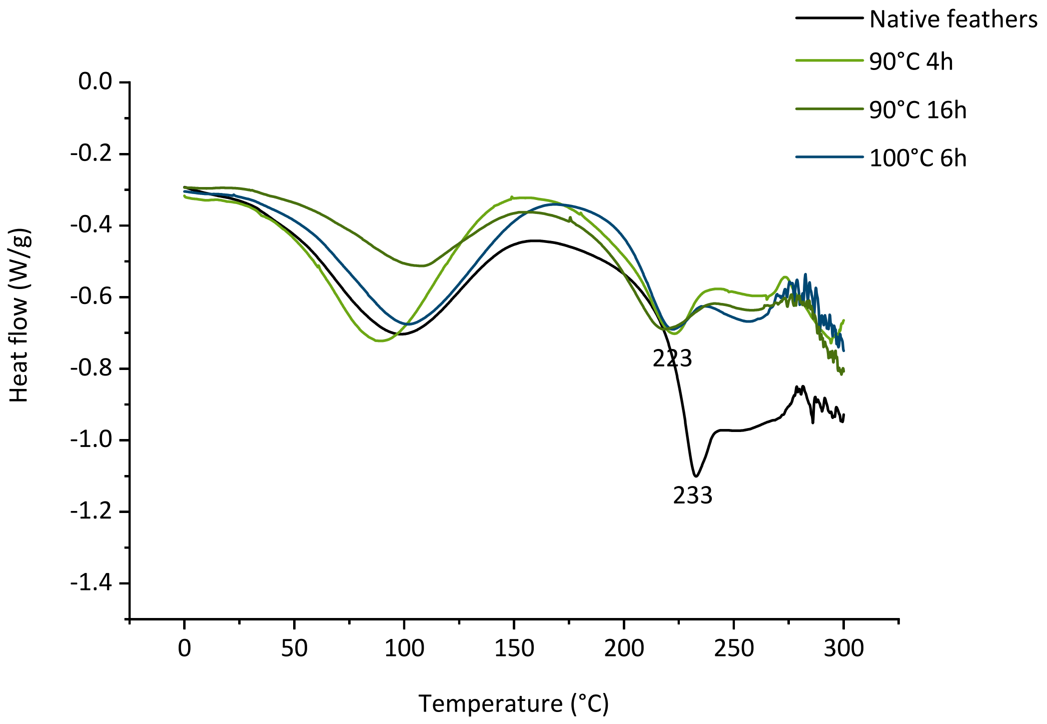

Fig. S7 Dynamic DSC thermograms at 10 °C/min for raw material and regenerated keratin samples.

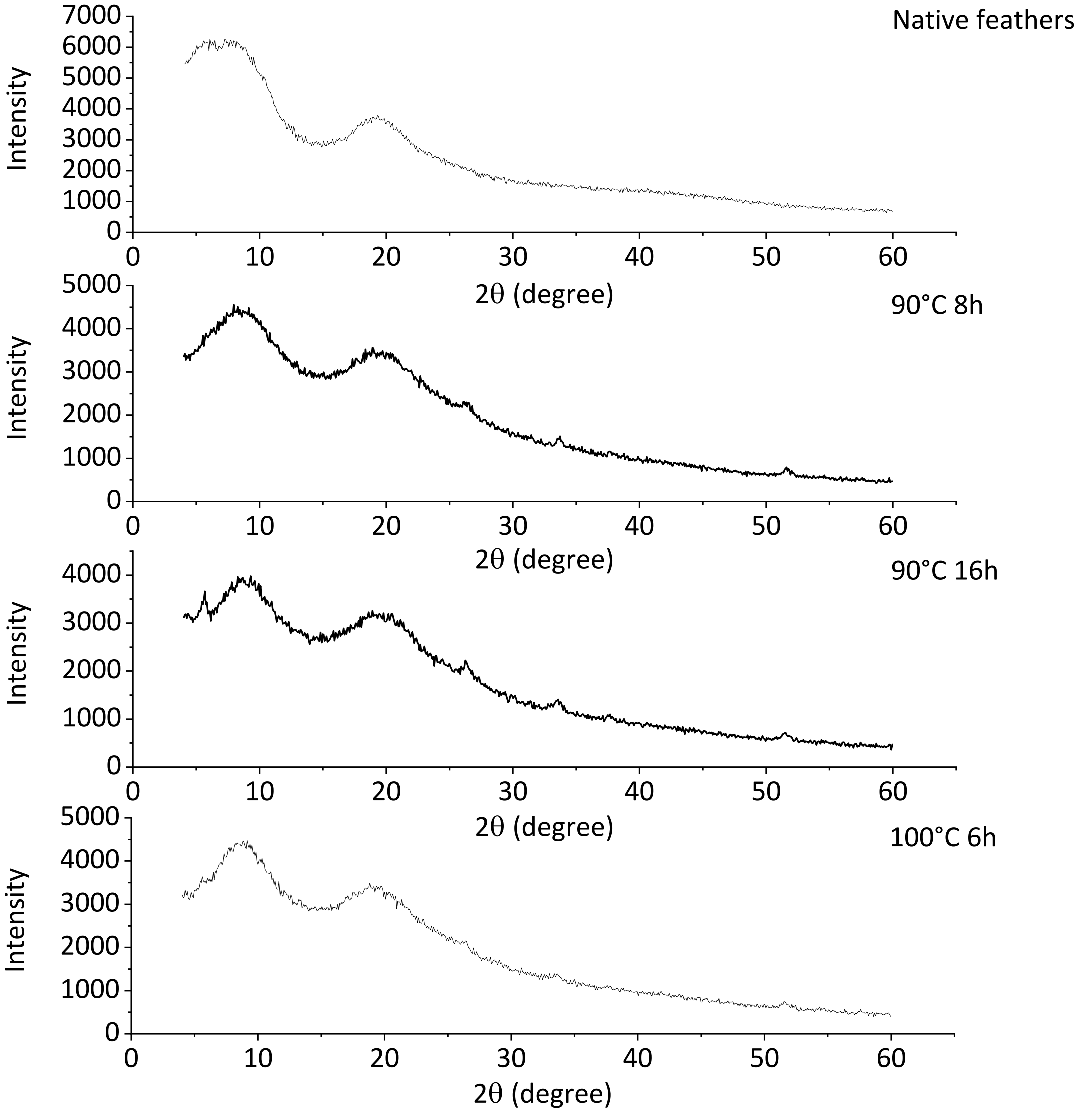

Fig. S8 XRD patterns of the raw material and regenerated keratin samples.

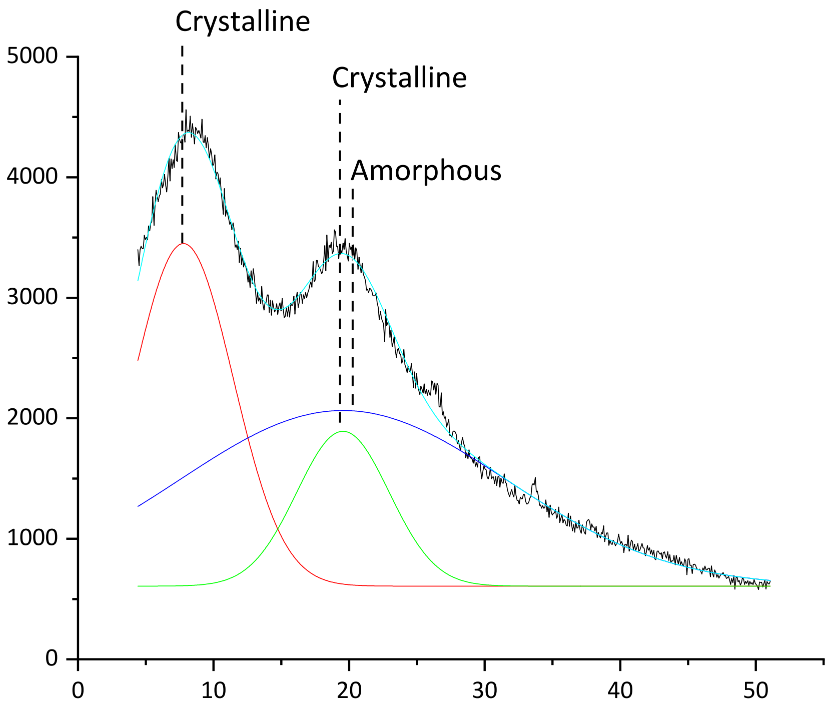

Fig. S9 Peak fitting in XRD pattern.
